# Endodontic and Surgical Management of Root Perforation Caused by Intermaxillary Fixation (IMF) Screw Placement: A Case Report

**DOI:** 10.7759/cureus.71716

**Published:** 2024-10-17

**Authors:** Mohammed Alhumaid, Yousra Alkhairallah, Abdullmajeed Altokheus, Lama A Alzahrani, Abdulaziz Altahtam

**Affiliations:** 1 Endodontics, Prince Sultan Military Medical City, Riyadh, SAU; 2 General Dentistry, Prince Sultan Military Medical City, Riyadh, SAU

**Keywords:** apical surgery, dental trauma, endodontic management, endodontic microsurgery, root perforation

## Abstract

The use of fixation screws is a valuable tool in the clinical practice of oral and maxillofacial surgeries. One potential complication of screw placement for fracture fixation is root damage during surgery. In some cases, this damage can further lead to irreversible pulp damage, which requires endodontic and/or surgical intervention.

The present report describes a case of iatrogenic root perforation during intermaxillary fixation (IMF) screw placement, leading to pulp necrosis. Despite the complication, the root damage was effectively managed through non-surgical root canal treatment using calcium silicate root filling material, followed by surgical apicectomy and retrograde fill. The patient was asymptomatic at a nine-month follow-up, and radiographic examinations showed signs of initial bone healing at the surgical site. After the successful management of the root perforation, the patient was referred to the prosthodontics department for the placement of the final restoration.

It is important to understand the potential damage that may occur with screw-root contact and how it can be avoided or minimized. This report highlights the challenges and considerations in managing such cases and emphasizes the importance of using IMF screws with caution and the need for regular follow-ups in instances where tooth root damage has been confirmed.

## Introduction

Dental trauma often necessitates surgical interventions, such as plates or screw placement for fracture stabilization. However, these procedures can inadvertently lead to complications, including root damage, which significantly impacts the prognosis of the affected tooth. Root damage can result in multiple complications such as ankylosis, pulp necrosis, and apical periodontitis [1]. Such conditions can arise when bacteria infiltrate the pulp through damaged dentin, leading to inflammation and infection. There are several key factors contributing to root damage during screw placement, including proximity to the roots, screw length, insertion angle, and the technique used by the clinician [2,3]. Intermaxillary fixation (IMF) screws have become a popular alternative to arch bars or eyelet wires due to their ease of use, compatibility with plating systems, and ability to reduce buccal and lingual soft tissue trauma [4]. A study aimed to evaluate the efficacy of IMF screws compared to modified arch bars in patients with maxillomandibular fixation requirements and found that patients treated with IMF screws demonstrated better oral hygiene maintenance as well as less operating time and complications [5]. However, the use of IMF screws still carries potential risks, including screw loosening, iatrogenic dental injuries, and postoperative malocclusion [6,7].

Conventional root canal treatment alone can sometimes be insufficient; various tooth-related factors, such as perforations or resorptions, may often necessitate additional apical surgery or apicectomy. Over the years, endodontic surgery has advanced into what is now known as endodontic microsurgery; this evolution includes the use of microscopes or loupes, ultrasonic root-end preparation, and the use of calcium silicate materials [8]. Similar to the current reported case, the mental and inferior alveolar neurovascular bundles are often found near the surgical sites of the mandibular molars and premolar area. To ensure a successful procedure, it is important to utilize radiographic assessments such as cone-beam computed tomography (CBCT) imaging, along with thorough treatment planning [9].

This report describes the endodontic management of iatrogenic root perforation of a mandibular left first premolar, caused by the insertion of an IMF screw.

## Case presentation

**Figure 1:**
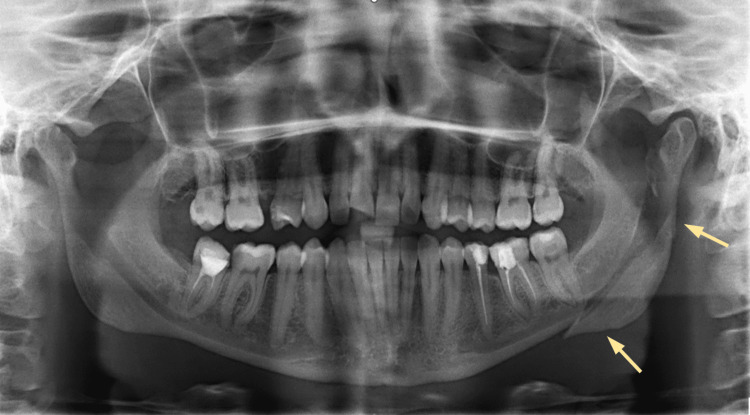
A preoperative panoramic radiograph (orthopantomogram (OPG)) showing fracture of the left mandibular subcondyle and ramus

A 34-year-old female presented to the emergency department following trauma caused by loss of consciousness. All diagnostic investigations of the patient were found normal, except for the CT scan of the facial bones and panoramic imaging, which showed a comminuted, mildly displaced fracture of both the left mandibular subcondyle and the ramus, as seen in Figure 1.

The treatment plan included open reduction and internal fixation with plates and screws for the comminuted left mandible and subcondylar fracture through a retromandibular approach. During the surgery, one of the IMF screws planned to be positioned between tooth #33 and #34, was accidentally drilled into the root of tooth #34, causing a perforation. The IMF screws were removed six weeks after the surgery.

**Figure 2:**
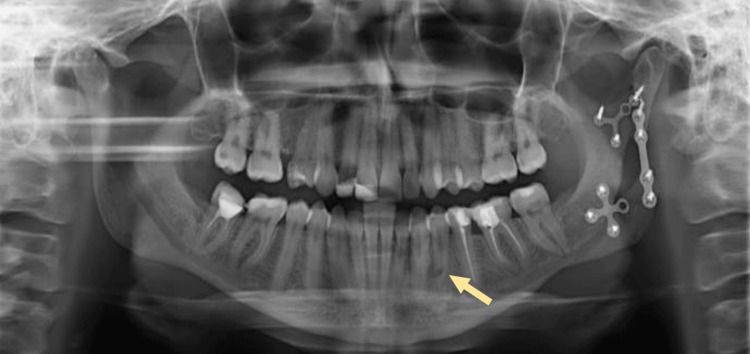
A panoramic radiograph (orthopantomogram (OPG)) taken nine months after the removal of the IMF screws showing perforation related to the root of tooth #34

**Figure 3:**
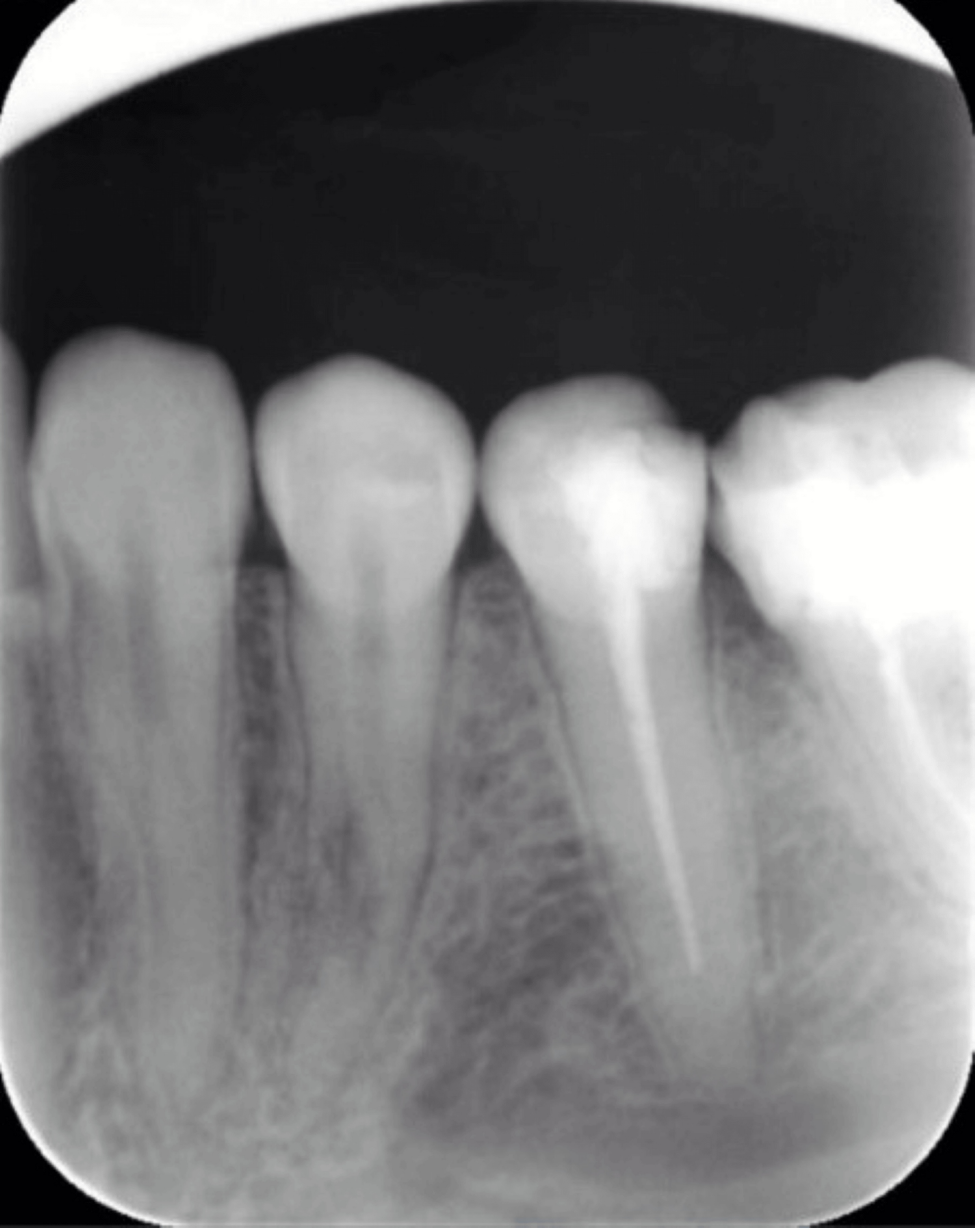
A periapical radiograph (PA) of tooth #34 showing an irregular radiolucency related to the root

**Figure 4:**
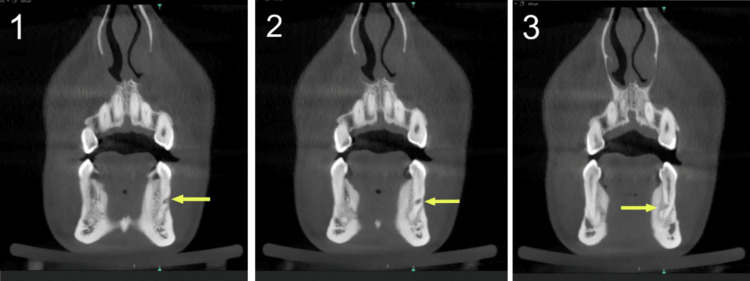
A coronal view of the cone-beam computed tomography (CBCT) image showing perforation of tooth #34 root, starting from the buccal area at the middle third of root (1) and proceeding in a more apical direction (2,3)

**Figure 5:**
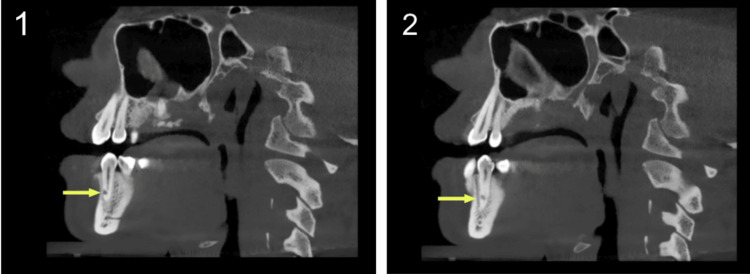
A sagittal view of the cone-beam computed tomography (CBCT) image showing perforation of the root of tooth #34, starting from the buccal area (1) and moving lingually (2)

**Figure 6:**
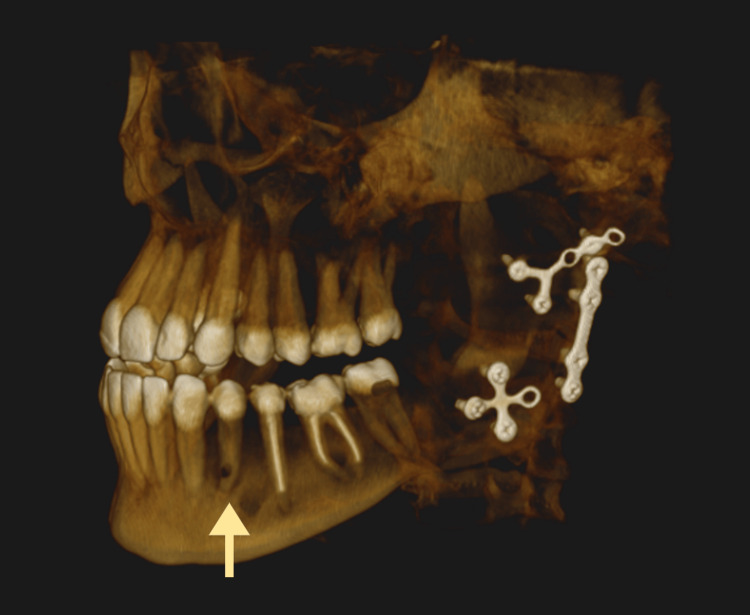
A 3D reconstruction of the cone-beam computed tomography (CBCT) image showing perforation of the root of tooth #34

Nine months following the IMF screw removal, the patient returned for a follow-up visit where she reported pain during mastication and reoccurring localized swelling. A panoramic radiograph (orthopantomogram (OPG)) showed a round radiolucency in the root of tooth #34 (Figure 2). A periapical radiograph (PA) was taken, which confirmed the presence of a perforation (Figure 3). A CBCT image was taken to confirm the location and extent of the perforation, which revealed a perforation in the middle third of the root of the mandibular left first premolar, extending buccolingually through the root canal space of the tooth (Figures 4-6).

**Figure 7:**
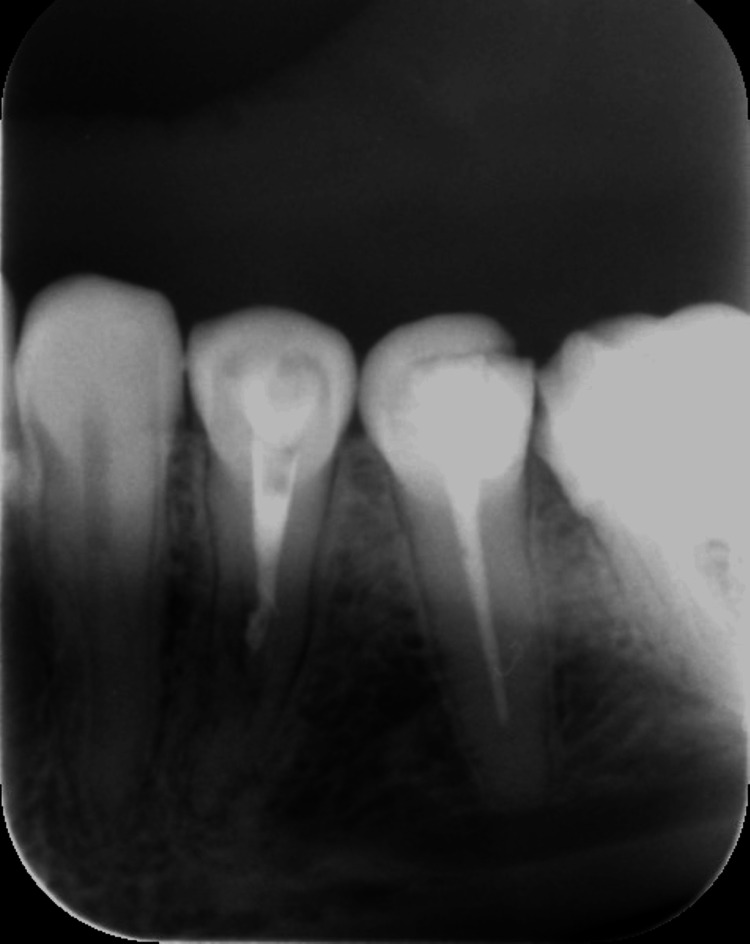
A periapical radiograph (PA) of tooth #34 showing calcium silicate cement up to the level of perforation, where the canal was blocked due to bone formation

The patient was then referred to the endodontic department, where the diagnostic examinations revealed pulp necrosis with symptomatic apical periodontitis. The endodontic treatment plan included conventional root canal treatment, followed by apicectomy. Under rubber-dam isolation and after canal negotiation with small K-files (sizes 8 and 10), the canal was blocked at the level of the perforation due to bone development in that area. Instrumentation and irrigation with 5.25% sodium hypochlorite were done until the level of blockage, followed by orthograde filling with only calcium silicate cement (total fill bio-ceramic root repair material), which was placed into the canal using a ready-to-use syringe (Figure 7).

**Figure 8:**
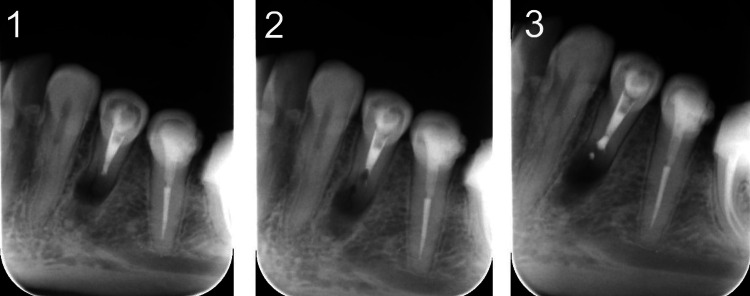
A periapical radiograph (PA) of tooth #34 after the apical surgery (1), after cavity preparation (2), and retrograde fill with IRM (3) IRM: intermediate restorative material

**Figure 9:**
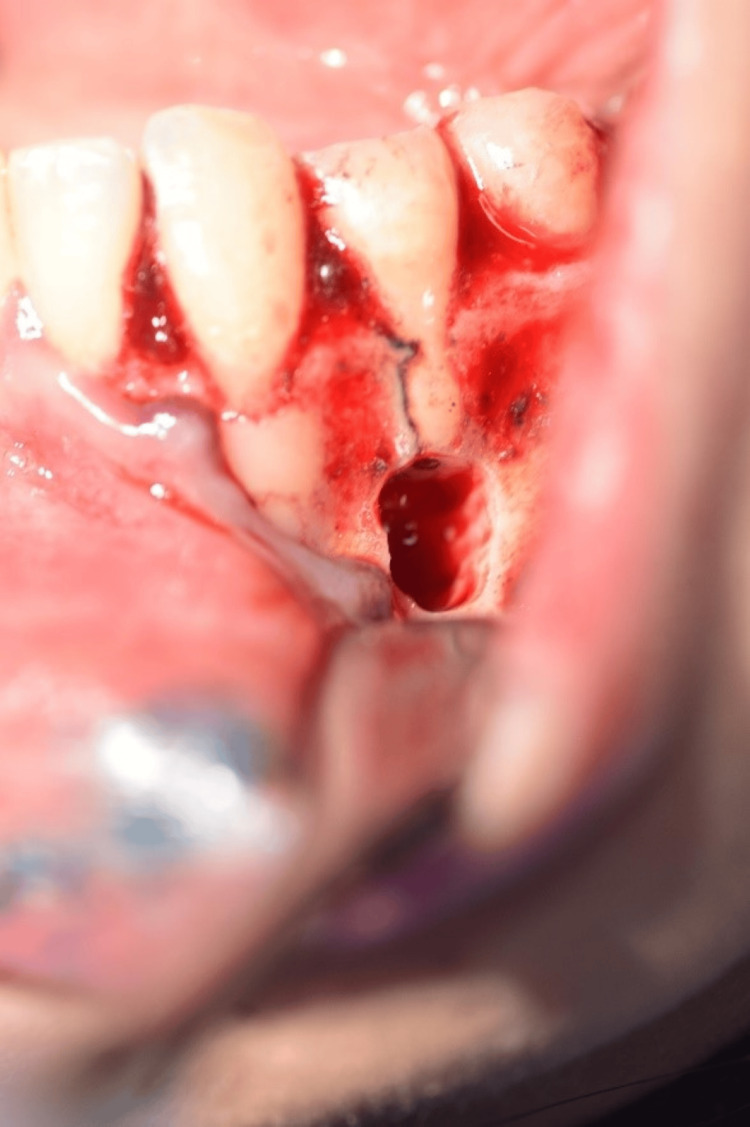
Clinical photo during the apical surgery, after resecting the apical part of the root of tooth #34

As for the apicectomy, the treatment plan was explained and discussed with the patient, including all the details of the surgical procedure, such as the risks, benefits, alternatives, and prognosis. Informed consent was then obtained from the patient. After local anesthesia administration, using three capsules of lidocaine with epinephrine 1:100,000, the patient was instructed to use a 0.2% chlorohexidine gluconate mouthwash. A full mucoperiosteal flap was then raised, extending from tooth #33 to #36 with the distal releasing incision distal to #36. Apical root resection was done for tooth #34, followed by retrograde preparation using ultrasonic tips and retrograde filling with intermediate restorative material (IRM) (Dentsply Intermediate Restorative Material, Dentsply Sirona, Charlotte, NC) (Figures 8, 9). The incision was closed with simple interrupted sutures.

**Figure 10:**
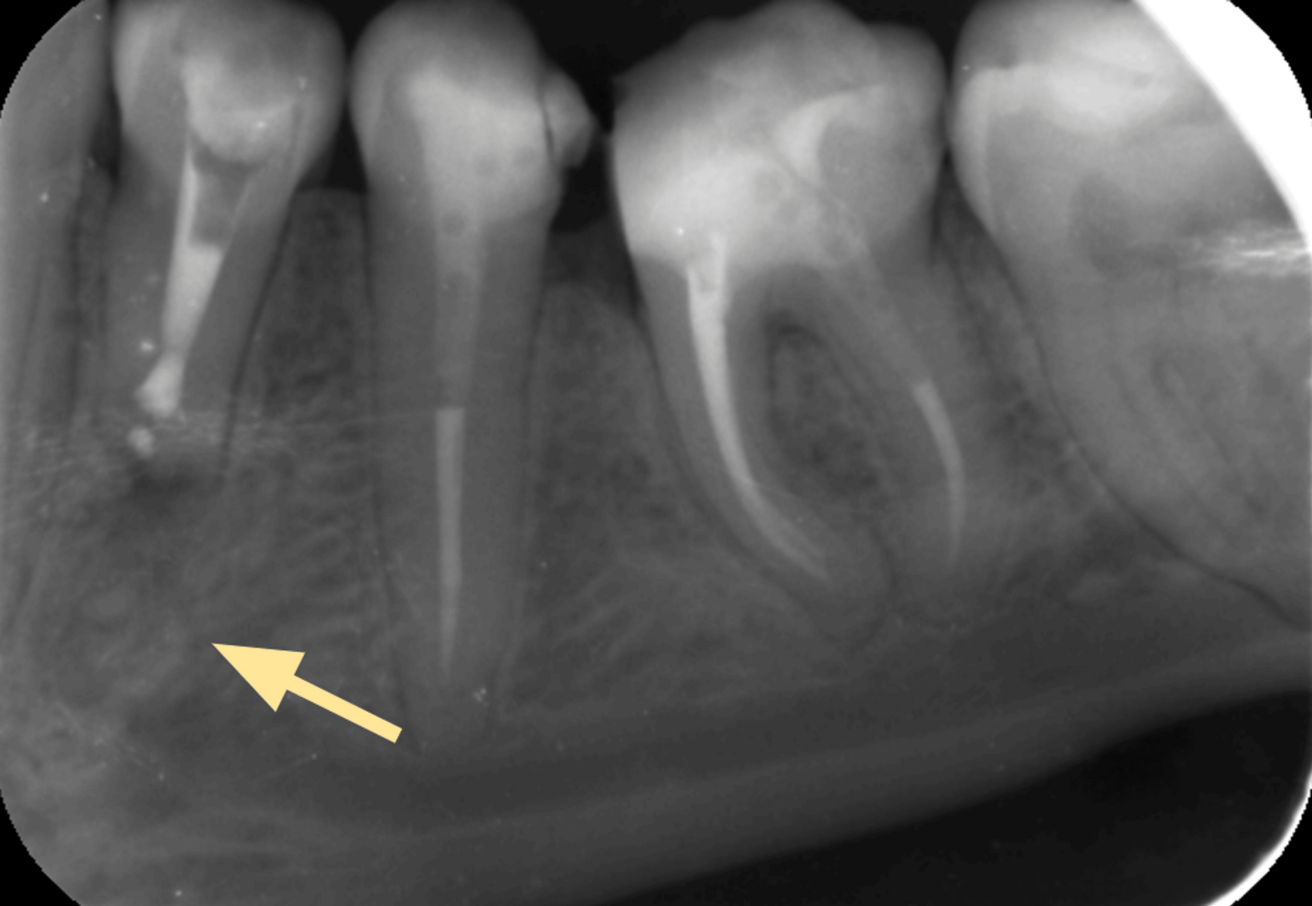
A periapical radiograph (PA) of tooth #34 showing initial healing and bone formation at the apical surgery site

**Figure 11:**
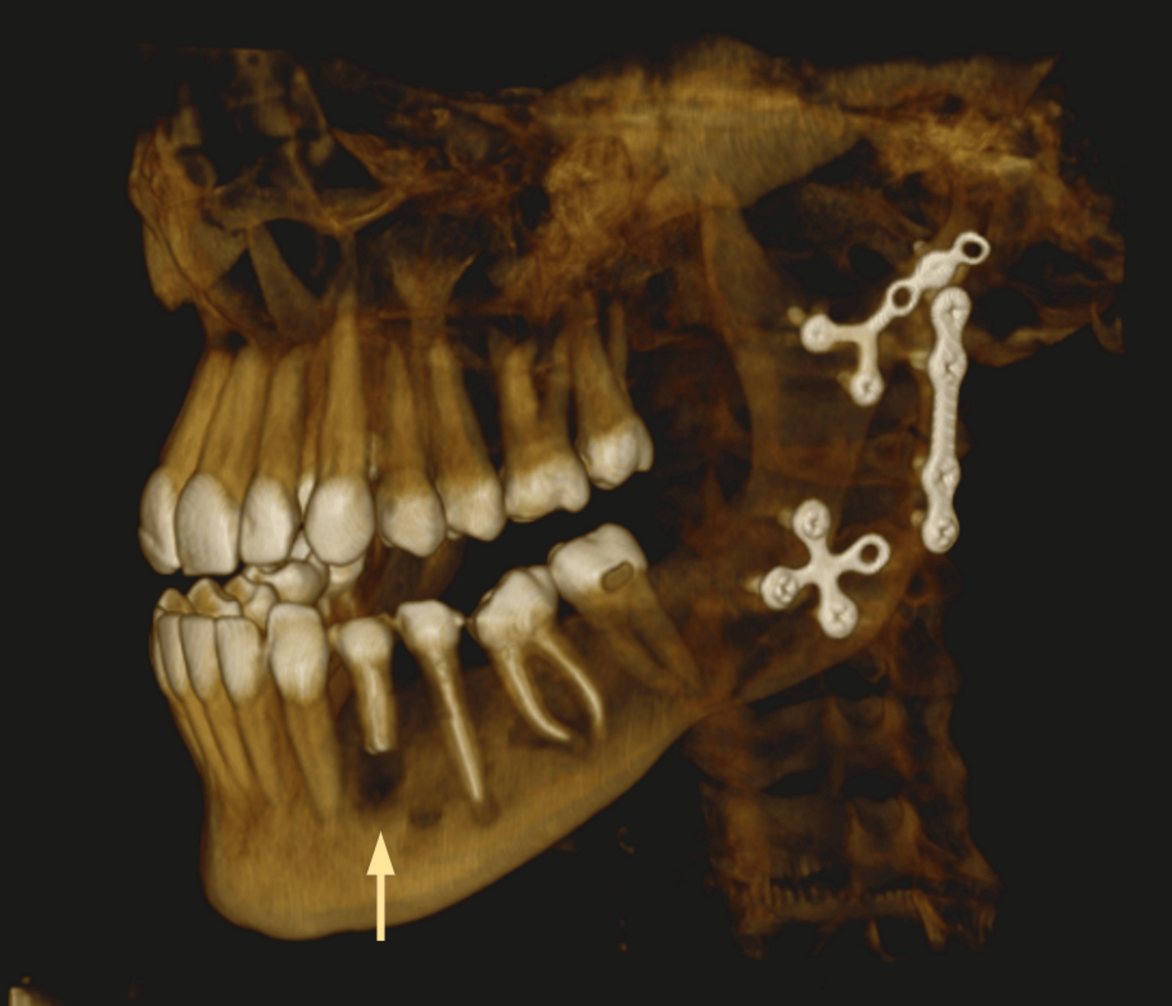
A 3D reconstruction of the cone-beam computed tomography (CBCT) image taken seven months after the apical surgery

Afterward, at the seven-month follow-up following the apical surgery, after the clinical examinations, the patient was found asymptomatic, and both PA and CBCT images revealed initial bone healing (Figures 10, 11). Finally, the patient was referred to the prosthodontics department for the final coronal restoration.

## Discussion

Effective management of mandibular fractures relies on achieving proper reduction, restoring normal occlusion, and ensuring adequate fixation. Intermaxillary fixation is a routinely used procedure in the management of mandibular fractures. In this case report, the patient presented with a comminuted fracture of the left mandibular ramus and subcondyle, which was managed with IMF screws and plates. Intermaxillary fixation is done in various ways, such as with arch bars or eyelet wires. However, the use of these techniques often requires considerable time for both application and removal. These methods also carry significant risks, including the possibility of the sharp wires perforating the surgeon's gloves, leading to “needle-stick” injuries. Furthermore, tightening of the wires around the teeth may cause ischemic necrosis of the surrounding mucosa, which may lead to tooth extrusion or loss of vitality. Additionally, maintaining effective oral hygiene can also be challenging with these methods [10]. In this case report, the complication reported was perforation of the root of the lower left first premolar. A retrospective study involving the placement of 880 screws found that iatrogenic root damage is one of the most significantly noticed complications. Similar to this case report, a retrospective study found that at the time of removal of 18 screws, two premolars presented with perforations through the roots extending into the pulp cavity, which were subsequently managed with endodontic treatment [7]. Several articles have reported the incidence of root damage during IMF procedures; in a study on 423 cases, dental injury occurred after insertion of 30 screws out of 2,100 screws [11]. Another study detected 16 (0.5%) dental injuries in 13 out of 366 patients in a mixed collective, including trauma treatment and orthognathic surgery [12]. In addition, a study analyzing the application of 1,067 screws found that 133 radiological tooth root injuries were observed (12.5%). Notably, the majority of these injuries occurred in the lower jaw, accounting for 62.4% of the total injuries reported. This finding is consistent with the current case report [13].

Choosing the appropriate treatment plan for tooth root damage depends on several factors, including the size and location of the perforation, the tooth vitality status, and the condition of the surrounding periodontium. In cases where the root is superficially injured and there is no pulpal involvement, the injury may heal by the deposition of cellular cement once the contact with the screw has been broken. However, in cases of extensive damage to the root that involves the pulp tissue and causes the tooth to lose its vitality, endodontic and surgical treatment are usually needed [1]. Therefore, it is crucial to perform a thoughtful evaluation of the injury to determine the best treatment approach, whether it is nonsurgical or surgical endodontic treatment or a combination of both.

In the current case report, the tooth was diagnosed as necrotic with symptomatic apical periodontitis. Therefore, the perforation was first managed by non-surgical endodontic treatment, and the material of choice was bio-ceramic (BC) sealer. The advantages of this material have been mentioned in multiple articles, owing to their unique physicochemical and biological properties. These materials exhibit exceptional sealing capabilities due to their interactions with the surrounding environment, as well as high biocompatibility, ease of handling, and high antimicrobial properties [14-16]. Like the present case report, two case reports published in 2021 demonstrated that the use of bio-ceramics for the repair of lateral canal perforations resulted in favorable outcomes, as observed during a one-year follow-up period. It was also concluded that bio-ceramics could serve as an effective therapeutic alternative to mineral trioxide aggregate (MTA) [17]. 

The next phase in the management of this case report was apical surgery to resect the apical part of the root, followed by retrograde filling with IRM. Clinicians must pay careful attention to the surrounding vital structures at the surgical site. In this case report, the mental foramen was in close proximity to the affected root. In order to prevent injury to the mental nerve, effective pre-surgical planning is necessary, including preoperative measurements from the periapical radiographs and CBCT imaging [8]. Additionally, the concept of endodontic microsurgery was followed in this case report to ensure a minimally invasive procedure. This included keeping the osteotomy site as small as effectively possible, along with the use of magnification and ultrasonic tips for root end preparation. A study on the long-term prognosis of endodontic microsurgery showed that the long-term prognosis for endodontic microsurgery demonstrated high success rates, ranging from 78% to 91% over follow-up periods of two to 13 years. This approach can be reliable when using modern surgical techniques and biocompatible, bioactive root-end filling materials [18]. A retrospective analysis of 81 teeth treated with apical surgery concluded that 74 teeth showed successful clinical and radiographic healing at one-year follow-up, resulting in a success rate of 91.4%. These results suggest that apical surgery, in conjunction with retrograde root-end filling, is a reliable therapeutic option for tooth preservation [19]. Another retrospective study done on 1,347 apicoectomies to compare between IRM and MTA as apical barriers concluded that the success rate of apicoectomies was 71%, regardless of the type of material used [20]. In regard to the current case, at seven months follow-up, the periapical radiograph shows signs of initial bone healing (Figure 9). The patient was symptom-free and satisfied, indicating a favorable outcome in the overall management of the current case.

In the present case report, the root perforation associated with tooth #34 may be due to the fact that in the lateral aspects of the jaw, optimal drill positioning could be difficult to achieve and, in consequence, carries a higher risk of dental root damage [21]. Moreover, the higher incidence of dental root damage observed in the lateral aspects of the jaws may be related to the more complex anatomical structures in these areas. In contrast, the anatomy of anterior tooth roots is generally more predictable due to their relatively straight orientation. The variations in root morphology, tooth positioning, and interradicular distances can influence the accuracy of screw placement, potentially resulting in such complications [22].

Recommendations to avoid complications of IMF screws suggest that particular attention should be taken regarding the surgeon’s positioning during screw insertion, and a direct view of the insertion site is essential to prevent angulation errors [21]. A precise approach during the drilling of the bur hole is crucial. The screw should be inserted at a steady speed and should not be forced if resistance is encountered, as this may suggest contact with the tooth roots [23].

## Conclusions

This case report documents the successful management of a root perforation due to IMF screw insertion through a combination of root canal treatment and apical surgery. The patient remained asymptomatic throughout the follow-up period, and radiographic examinations indicated initial signs of bone healing at the surgical site. The results and supporting literature suggest that this approach can be effective in managing similar cases.

For some cases, such as perforations and resorption, conventional root canal treatment alone can be insufficient, and additional procedures such as apical surgery may be required. Endodontic surgery has significantly advanced with the incorporation of ultrasonic tips, magnification, and bioactive materials. These advancements have not only improved the precision of surgical procedures but have also led to more favorable outcomes. Finally, it is essential to be aware of the potential harm that can arise from screw-root contact and how it can be prevented or reduced. This report stresses the need to use IMF screws with caution along with thorough treatment planning and to ensure regular follow-ups once tooth root damage has been confirmed.
